# The Association between Greenness and Urbanization Level with Weight Status among Adolescents: New Evidence from the HBSC 2018 Italian Survey

**DOI:** 10.3390/ijerph19105897

**Published:** 2022-05-12

**Authors:** Valeria Bellisario, Rosanna Irene Comoretto, Paola Berchialla, Emanuele Koumantakis, Giulia Squillacioti, Alberto Borraccino, Roberto Bono, Patrizia Lemma, Lorena Charrier, Paola Dalmasso

**Affiliations:** 1 Department of Public Health and Pediatrics, University of Torino, Via Santena 5 bis, 10126 Torino, Italy; valeria.bellisario@unito.it (V.B.); rosannairene.comoretto@unito.it (R.I.C.); emanuele.koumantakis@unito.it (E.K.); giulia.squillacioti@unito.it (G.S.); alberto.borraccino@unito.it (A.B.); roberto.bono@unito.it (R.B.); patrizia.lemma@unito.it (P.L.); lorena.charrier@unito.it (L.C.); paola.dalmasso@unito.it (P.D.); 2 Post Graduate School of Medical Statistics, University of Torino, Via Santena 5 bis, 10126 Torino, Italy; 3 Department of Clinical and Biological Sciences, University of Torino, Regione Gonzole 43, 10043 Orbassano, Italy

**Keywords:** adolescence, health promotion, normalized difference vegetation index (NDVI), obesity, physical activity, public health, urbanization

## Abstract

Recent studies have examined how the environment can influence obesity in young people. The research findings are conflicting: in some studies, green spaces have shown a protective association with obesity and urbanization has turned out to worsen this condition, while other studies contradicted these results. The aim of the study was to examine the relationships between greenness, urbanization, and weight status among Italian adolescents. Student data (11–13 years old) on weight and height, physical activity (PA), and demographic characteristics were extracted from the 2018 Health Behaviour in School-aged Children (HBSC) survey in Piedmont, Northwest of Italy. Data on Normalized Difference Vegetation Index (NDVI) and urbanization were obtained from satellite images and the National Institute of Statistics (ISTAT). A multilevel regression model was used to assess the association between NDVI, urbanization, and obesity, controlling for PA. Students living in greener areas reported a lower likelihood of being obese [OR = 0.11, 95% CI 0.02–0.56, *p* = 0.007], while students living in areas with a higher level of urbanization showed a significantly increased risk of obesity [OR = 2.3, 95% CI:1.14–4.6, *p* = 0.02]. Living surrounded by higher amounts of greenness and lower levels of urbanization may positively influence health status through lower risk of obesity among youth.

## 1. Introduction

Green spaces can provide a safe, convenient, and attractive place for physical exercise, whose health benefits have been well documented (cardiorespiratory fitness improvement, weight control, reduction in anxiety and depression, among others) [1,2,3,4]. However, several studies showed contrasting results when assessing the association between green spaces and weight-related issues among young people, particularly for overweight and obesity [5,6,7,8,9]. Some studies showed that the availability of green areas appeared to influence obesity and promote physical activity (PA), although these associations could be partially explained by other factors, such as family socioeconomic status (SES) [10,11,12,13]. Conversely, other studies found negative associations between green spaces and PA [14,15,16]. In fact, although greener spaces could offer opportunities for recreation and sports, these areas may also imply longer walking distances to services and other recreational spots, which could also favor cars or public transport utilization [17,18,19,20]. Several reasons have been given to explain these conflicting results: different techniques in quantifying green spaces concentrations; the environmental characteristics of the home and school neighbourhood; the surrounding obesogenic environment profile, such as fast-food restaurants on the path to green spaces; and disadvantaged rural areas [21,22,23,24].

Furthermore, the dramatic growth of urban areas and the consequent loss of green spaces can affect health and PA levels, limiting the opportunity to be physically active and increasing sedentary behaviours and energy imbalance intake [21,25,26].

The study aims to investigate whether and how the environmental context is associated with the weight status of adolescents. Although evidence of the association between weight status and exposure to neighbourhood greenness has been reported, only a few studies have evaluated the association simultaneously considering greenness and urbanization levels [26,27]. Thus, the primary objective was to examine the relationship between greenness, urbanization, and weight status in a regionally representative sample of Italian adolescents.

## 2. Materials and Methods

### 2.1. Study Population

The Health Behaviour in School-aged Children (HBSC) is a World Health Organization collaborative cross-national study to investigate the health and well-being of adolescents in Europe and North America. Data collection follows a standard methodology outlined in the HBSC protocol [28,29]. Data are collected every four years among representative samples of 11-, 13-, and 15-year-old students through a self-filled standardized questionnaire (Health behaviour in school-aged children. World Health Organization collaborative cross-national survey (www.hbsc.org)). Italy joined the HBSC international network in 2001 and has so far carried out five data collections, the last three conducted in all national regions, ensuring their representativeness [30]. A detailed description of the aims, theoretical framework, and protocol of the international HBSC study and its Italian component can be found elsewhere [28,29].

In this study, we used data from the 2018 HBSC Italian survey. We extracted information for the Piedmont Region, the second largest region of Italy, located in the Northwestern part of the country. A representative sample of students was drawn from 63 middle schools (overall response rate: 97.4%). Since environmental measures were collected at the school level and because 15-year-old students can attend schools that may be far from their home district, we limited the analysis for this study purposes to 11- and 13-year-old subjects.

### 2.2. Measurements

#### 2.2.1. Individual-Level Data

Individual-level data on demographic characteristics (age, gender, and socioeconomic status), Body Mass Index (BMI), and physical activity were obtained from the 2018 HBSC survey.

Gender and age. Students were asked to indicate whether they were a boy or a girl and their month and year of birth.

Socioeconomic status (SES). The SES of the students’ families was assessed according to the Family Affluence Scale (FAS), a reliable indicator of family wealth [31,32,33]. The scale consists of six items: family car ownership, whether adolescents have their own bedroom, number of holidays trips taken in the last year, number of computers owned by the family, dishwasher ownership, and number of bathrooms in the home. The obtained score (0–13) was recorded in a 3-point ordinal scale according to low (0–6), medium (7–9), and high (≥10) SES.

Obesity. Self-reported height and weight were used to calculate BMI (kilogram per square meter). Students’ body weight status was assessed according to the International Obesity Task Force [34]. The present study categorized the students as overweight or obese (OwO) and not overweight or obese (not OwO).

Moderate-To-Vigorous Physical Activity (MVPA). Frequency of physical exercise was assessed with a single item, originally developed by Prochaska J.J. et al. [35] and evaluated as a recommendable brief surveillance measure [36,37]: students reported the number of days over the past week during which they were physically active for a total of at least 60 min. Participants were prompted to list activities, including running, dance, and sporting activities. Response options ranged from 0 to 7 days.

#### 2.2.2. Environmental-Level Data

All 63 middle schools sampled were geolocalized, and then fixed buffers (radius 1500 m) around each school were built for NDVI calculations. We considered schools as a proxy of the home address range, assuming that adolescents aged between 11 and 13 years old are supposed to attend school within their home district and do not have an independent mobility pattern [38,39].

Normalized Difference Vegetation Index (NDVI). Greenness exposure was assessed using the NDVI index [38,40], a commonly environmental index that quantifies the vegetated biomass, considering that chlorophyll in healthy vegetation mostly reflects the near-infrared band (NIR) (0.7–1.1 µm) compared to the other wavelengths of the light spectrum and, at the same time, strongly absorbs visible light (RED) (0.4–0.7 µm). NDVI is calculated as the ratio of the difference between the NIR and the RED bands to their sum, and ranges between −1 and 1, where higher positive values indicate more dense green vegetation. Consequently, a reasonable approach to quantify greenness exposure level is the characterization of the vegetated areas falling in a buffer zone surrounding subject houses [4].

In this study, NDVI was derived from a cloud-free satellite summer image (Landsat 5, resolution 30 m × 30 m) [41], referring to the same month and year of the collection of HBSC data (May 2018). Greenness exposure was calculated for all participants within the fixed buffer (radius 1500 m) around their school address.

Urbanization. Statistics by the degree of urbanization provide an analytical and descriptive lens on urban and rural areas. The EUROSTAT Degree of urbanization (DEGURBA) classifies municipalities according to the ecoregions identified, depending on clime physiographic and environmental factors [42]. This approach, adopted in Italy by the Italian National Institute of Statistics (ISTAT), provides a hierarchical classification of the local administrative municipalities. Based on the share of the local population living in urban clusters and urban centres, it classifies local administrative units into three types of areas: cities (densely populated areas)/towns and suburbs (intermediate density areas)/rural areas (thinly populated areas) [42,43]. The final classification of the municipalities of the Piedmont region, where the schools of this study are located, provides the three different levels of urbanization described, implying both anthropometric and environmental factors [43].

### 2.3. Statistical Analyses

The demographic and environmental characteristics of the subjects were summarized as absolute and relative frequencies for categorical variables and as means (±SD) for continuous variables.

Due to the hierarchical structure of our data (2065 students nested into 63 schools), a multilevel regression model was performed to assess the association between OwO, as the dependent variable; NDVI, included in the model as a continuous variable; and urbanization level, controlled for age group, gender, SES and MVPA. Results were reported as odds ratios (ORs) with 95% confidence intervals (CIs). All analyses were carried out using the STATA 16.1 software (StataCorp LLC: College Station, TX, USA).

## 3. Results

Table 1 reports individual and environmental data by age group. Overall, 2065 students were included in the analysis. Among these, 52.4% were males, and 51.6% were 13 years old. Moreover, 20% percent of boys and 11% of girls were OwO: 16.4% and 15.3% among 11 and 13 year olds, respectively. SES and MVPA frequencies showed similar distributions between the general sample and age groups. Regarding environmental data, about 22% of the students lived in the highest urbanized level, 21.3% and 22.4% among 11 and 13 year olds, respectively. NDVI levels were similar among gender and age groups.

**Figure 1:**
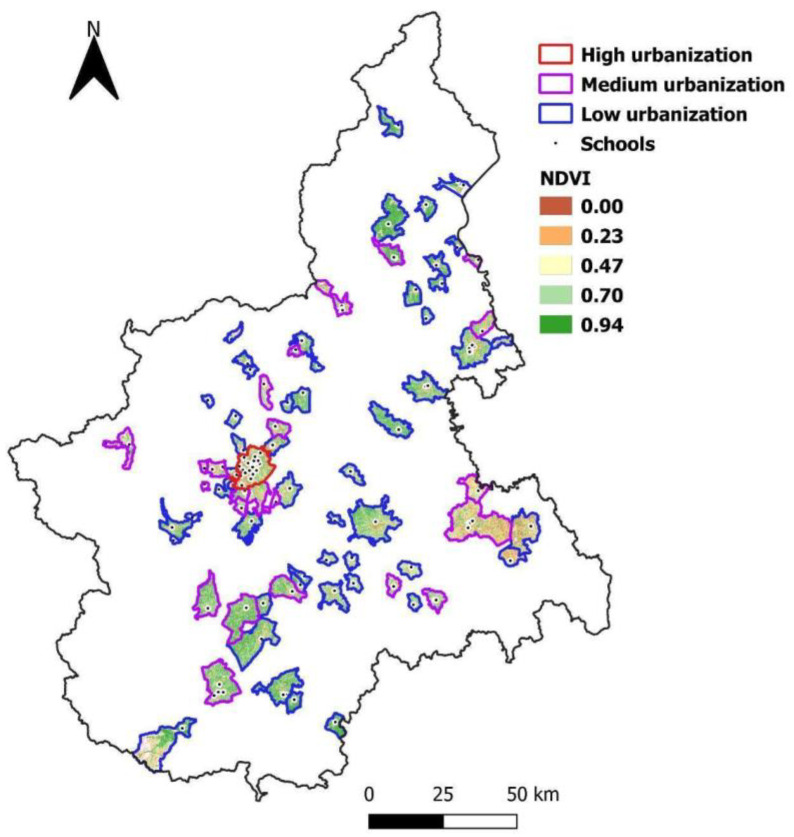
Geolocalization of 2018 HBSC schools in the Piedmont region and their results in NDVI and urbanization levels. Abbreviations: NDVI = Normalized Difference Vegetation Index. Urbanization classification: high = cities; medium = towns and suburbs; low = rural areas.

Figure 1 shows the Piedmont region with environmental measurements geo-referred to the schools analysed in the survey. In the map, different levels of NDVI, referring to the 1500 m buffer around schools have been represented: from low (0 = red) to high NDVI (0.94 corresponding to intense green). The figure also reported the urbanization classification referred to the municipalities (n = 63) where schools were located: blue and violet indicate low and medium urbanization levels, respectively, while Turin, the most urbanized city of Piedmont, is shown in red.

**Figure 2:**
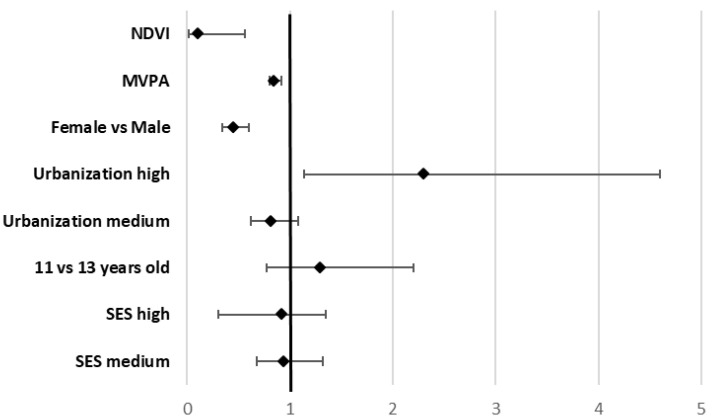
Multilevel regression model (OR and 95% CI) of OwO according to NDVI, urbanization levels, and physical activity, adjusted for gender, age group, and socioeconomic status. Abbreviations: OwO = Overweight and Obese; SES = Socioeconomic status; MVPA = Moderate-To-Vigorous Physical Activity; NDVI = Normalized Difference Vegetation Index.

Students who lived in areas with higher levels of greenness had less likelihood of being OwO than those with lower exposure to greenness in their surrounding environment [OR = 0.11 95% CI 0.02–0.56, *p* = 0.007] (Figure 2). On the contrary, students included in the highest level of urbanization showed a statistically significant association with OwO [OR: 2.3, 95% CI 1.14–4.6, *p* = 0.02]. Adolescents reporting higher physical activity were at a statistically significant lower risk of OwO [OR = 0.85, 95% CI: 0.8–0.92, *p* < 0.001]. Furthermore, female students showed a lower risk of OwO [OR: 0.45, 95% CI 0.34–0.6, *p* < 0.001]. No other statistically significant associations were found for medium urbanization level, age, and SES.

## 4. Discussion

Greenness has been explored as an environmental determinant of obesity due to its potential association with physical activity. Several studies showed that higher levels of greenness in the neighbourhood are associated with a lower likelihood of being overweight or obese [3,4,5,9]. In addition to greenness, this study also considered urbanization level as a further environmental determinant and potential confounders, such as SES. Our results attested to the protective effect of greenness on adolescent weight status [8,9,20,26]: adolescents living in areas with a lower level of urbanization and more green spaces showed a lower risk of being OwO. In line with previous studies [5,11,13], these results provided evidence that neighbourhood greenness has a protective effect on weight status, which could offer important implications for future research and policies.

Only a few studies have shown this strong positive association [10,11,21]. According to our findings, a cross-sectional study in China [44] found that higher school-based greenness levels were associated with lower BMI, waist circumference, and lower risk of overweight and obesity in children and adolescents. On the other hand, other studies did not reach these conclusions or statistical significance [21,22,23,24]. These conflicting results may be due to heterogeneous populations, lifestyles, and greenness assessment techniques. In fact, the composition and configuration of urban green spaces are usually measured through landscape metrics [45], but the NDVI index has recently gained prominence in studies analysing green spaces and their relationship with health [46,47]. For example, among the different tools to describe neighbourhood and greenness exposure, the View Green Index (VGI), a series of street view images classified into colour categories, has been used in a Chinese study [5], but to the best of our knowledge, no one of these greenness assessment methods have ever been applied in Italy or used in studies with an adolescent population [5,46]. Nevertheless, to date, the methods for defining and measuring green spaces must be improved to estimate individual exposure to green spaces [25] accurately.

Furthermore, consistent with previous literature [7,9,10,25,48,49,50,51], our results showed that high urbanization levels increase the probability of being overweight or obese among adolescents. At the same time, increased PA is associated with a lower likelihood of OwO. Several recent studies have pointed out the link between the environment and some weight-related outcomes, demonstrating that children growing up in urban areas are more likely to be obese than those in rural areas in many countries, including high and low/middle-income countries [21,23,52,53]. Moreover, the steadily increasing prevalence of overweight and obesity in youth has been attributed to the obesogenic environment, which encourages high-energy intake by providing ready access to high-calorie foods, and limiting opportunities for physical activity training [54,55].

Many studies suggested several mechanisms by which the environment and PA are connected and could influence weight status, for example, by contrasting or increasing sedentary behaviour or creating an obesogenic environment [2,23,56,57]. However, these relationships between health outcomes, PA, and the neighbourood environment deserve to be deepened and further investigated in future research to better elucidate these associations, especially in children and youth.

Regarding the gender difference, girls seem to underreport their weight more than boys [58], which could be part of the explanation of our result.

## 5. Strengths and Limitations

This study has several strengths. First of all, using data from a national survey conducted following a standardized and validated protocol (HBSC, 2018 Italian survey) allows the analysis of a representative sample of adolescents. Furthermore, to our knowledge, this is the first study that investigates the association between greenness and BMI in Italy, providing specific data for exploring the relationship between greenness and level of urbanization with weight status in adolescence.

The cross-sectional design of the present study represents the main limitation, as it does not allow for assessing the causal direction of the associations, so the results should be interpreted cautiously. It is important considering that height, weight and physical activity levels are self-reported, and thus potentially biased. For example, BMI based on self-reported data can produce lower prevalence estimates of OwO than those based on actual height and weight measurements [59]. Furthermore, eating and obesogenic habits, such as fast food consumption or availability, were not investigated because the subjects showed similar diet variety. Geographic variability was not extensive because all the data came from the same area (the Piedmont region). Another limitation is that we measured only the exposure to greenness (NDVI) without specifying the vegetation type. Finally, we assessed greenness around the school (1500 m buffer), considering the school as a proxy of individual greenness exposition. This is a limitation because the association between greenness and health outcomes is stronger when environmental exposure is individually assessed.

## 6. Conclusions

Consistent with previous studies, these findings showed a protective association between greenness and weight status among adolescents, whereas high urbanization levels seem to increase the risk of being obese. Furthermore, physical activity is significantly associated with a lower risk of obesity in our sample. In conclusion, our findings support the idea that adolescents attending schools with higher levels of greenness are less likely to be obese than those attending schools in more urbanized areas. This evidence highlights the impact of greenness, level of urbanization, and PA on weight status, thus being of relevant importance for integrating preventive health strategies into urban designs to reduce obesity in youth. Existing data indicate that the association between greenness and weight status, especially in adolescence, is still poorly understood; therefore, further research is needed to understand the specific mechanism by which green vegetation and, more generally, the environment can interact with BMI and health. Finally, longitudinal studies are advisable to establish the causality and pathways of the association between neighbourhood and health outcomes and to design effective interventions and policies to prevent and control adolescent obesity. These measures can be more effective if they are planned in accordance with local cultures and behaviours in related fundamental Public Health research and policies.

## Figures and Tables

**Table 1:** Individual and environmental data by age groups.

Characteristics		11 Years Old n = 1000	13 Years Old n = 1065	Total n = 2065
Male	n (%)	522 (52.2%)	560 (51.6%)	1082 (52.4%)
SES n (%)	Low	245 (24.9%)	252 (24.1%)	497 (24.5%)
	Medium	484 (49.2%)	495 (47.2%)	979 (48.2%)
	High	254 (25.9%)	301 (28.7%)	555 (27.3%)
OwO n (%)	Male	88 (16.8%)	101 (18.1%)	189 (19.8%)
	Female	52 (10.9%)	45 (8.9%)	97 (11.3%)
	Total	140 (16.4%)	146 (15.3%)	286 (15.8%)
MVPA (0–7 days/week) mean ± SD	Male	3.7 ± 2.0	3.8 ± 2.0	3.7 ± 2.0
	Female	3.4 ± 1.9	3.1 ± 1.9	3.2 ± 1.9
	Total	3.5 ± 1.9	3.5 ± 2.0	3.5 ± 2.0
Urbanization n (%)	Low	216 (21.6%)	263 (24.7%)	479 (23.2%)
	Medium	571 (57.1%)	564 (52.9%)	1135 (54.9%)
	High	213 (21.3%)	238 (22.4%)	451 (21.9%)
NDVI	mean ± SD	0.53 ± 0.14	0.51 ± 0.15	0.52 ± 0.15

Abbreviations: BMI = Body Mass Index; OwO = Overweight and Obese; SES = Socioeconomic status; MVPA = Moderate- To- Vigorous Physical Activity; NDVI = Normalized Difference Vegetation Index.

## Data Availability

The data presented in this study are not publicly available according to the Italian HBSC data access policy.
